# Popliteal Aneurism: Cure by Flexion

**Published:** 1867-02

**Authors:** P. Sidney Jones

**Affiliations:** London


### SELECTIONS.

---

*Popliteal Aneurism: Cure by Flexion.* By P. Sidney Jones, M. D. London.

H. W., aged 31, a gentleman following pastoral pursuits, called upon me, on November 17, 1865, complaining of rheumatic pains in the right knee which had troubled him for several months. During the last few days the pain had become much worse, especially at night after walking a good deal. Is accustomed to take violent horse exercise, and a few months since, in jumping from a vehicle in motion, wrenched his right leg. Upon examination of his limb I discovered a pulsating swelling about the size of a small orange, occupying the popliteal space. The pulsation was expansile and tearing, synchronous with the pulse at the wrist, and completely arrested by pressure upon the femoral artery in the groin. The tumor could be nearly emptied by pressure, and felt to fill again when the pressure was removed. The skin over the tumor was quite healthy, and not adherent. The fingers could be passed down between the tumor and the muscles bounding the popliteal space, so as to grasp it. No bruit was perceptible.

Mr. Nathan, one of my colleagues at the Infirmery, saw the patient with me on the 19th, and confirmed the diagnosis. As the pulsation in the aneurism was completely checked by acute flexion of the knee, I determined to employ Ernest Hart's method, and accordingly on the 21st, carefully bandaged the limb from the toes to the groin, and bent the knee, so that the heel was about eight inches from the buttock, retaining it in this position was a figure of 8 bandage. In two days the heel was brought to within three inches of the buttock; but as this degree of flexion could not be borne for many hours, notwithstanding the use of morphia, he was allowed occasionally to extend the limb, and it was always relaxed somewhat at night.

On the fifth day I suspended over the thigh, by means of a small cord, a conical bag filled with 9 lbs. weight of small shot. Whenever the pain from the flexion became unbearable, the patient extended his leg and placed the conical end of the bag over the artery in Scarpa's triangle. This completely controlled the circulation as long as the bag was kept directly over the artery; but finding the patient could not manage this himself, and not having any competent assistant, it was desisted from after two days' trial. This plan appears to me, to have an advantage over digital compression, in that it is much less fatiguing to hold a bag steadily than to compress a vessel with the fingers, and hence fewer assistants will be needed.

On the nineteenth day (December 11) from the commencement of the flexion treatment, there was considerably less pulsation in the tumor; and over the front of the knee, which was a good deal swollen, and on each side of the patella small vessels could be felt pulsating.

On the twenty-seventh day (December 19) the pulsation had greatly diminished, and on the twenty-ninth day it ceased entirely. Nothing could be felt but a slight rippling over the centre of the tumor.

On the thirty-first day he was allowed to come down stairs.

On December 27 (thirty-fifth day) my notes are: "Tumor quite solid and free from pulsation; limps a good deal; still has some pain in the ham."

I saw no more of this gentleman until a month later (January 22, 1866), when he called upon me, complaining of severe pain in the left ham, which had begun to trouble him about ten days before. On the evening before calling on me, after walking about two miles, the pain was very severe, accompanied with violent throbbing. Upon examination I discovered a pulsating swelling, about the size of a walnut, situated in the course of the popliteal as it lies under the gastrocnemius muscle. It had all the characters of an aneurism, and a loud bruit could be heard over it. Circumstances prevented the patient from submitting himself to treatment until February 5, when the tumor was in much the same state. I bandaged and flexed the limb as with the other leg, and though flexion did not control the circulation in the aneurism so completely as in the first case, yet on the fourteenth day all pulsation had ceased in the tumor. A month later I saw this patient again. He was then quite well, though complaining of weakness in the right leg. Both aneurisms were then quite solid, and that in the right ham had very much diminished in size.

While under treatment for these aneurisms, this patient took morphia—sometimes as much as three grains in the day; and the knee, in which he at first complained of much pain, was rubbed with laudanum and oil. Towards the middle of the period of treatment he complained of severe pain in the shin-bone.—*Medical Times and Gazette, Sept.* 8, 1866.

